# Project-based learning in mathematics and science: a review of contributions, prevalence, and challenges

**DOI:** 10.12688/f1000research.170698.1

**Published:** 2025-10-10

**Authors:** Fidele Ukobizaba, Jean Francois Maniraho, Alphonse Uworwabayeho

**Affiliations:** 1 African Centre of Excellence for Innovative Teaching and Learning Mathematics and Science (ACEITLMS),, University of Rwanda College of Education (URCE), Kayonza, P.O Box 55 Rwamagana, Rwanda

**Keywords:** Contributions of project-based learning, challenges, Mathematics and Science education, skills development, prevalence of project-based learning

## Abstract

The integration of project-based learning (PjBL) in Mathematics and Science has received much attention because of its potential to engage students and expose them in real-world problem-solving. However, research examining the contributions, prevalence, and challenges of Project-Based Learning in mathematics and science remains limited. This review employed a systematic review approach, with articles systematically retrieved from multiple academic databases to investigate the contributions, prevalence, and challenges of PjBL in Mathematics and science education. Thus, 202 articles downloaded from Google Scholar, Academia, Search 4 Life, Scopus, and Web of Science databases. Through the filtering process, 20 articles fell into the study’s scope and were considered and used for analysis. The results from the reviewed studies showed that project-based learning contributes to enhancing students’ engagement, creativity, communication, and conceptual understanding in Mathematics and Sciences. Also, the reviewed literature showed that PjBL dominates in Mathematics and Physics with the highest prevalence of 35% each in applying PjBL pedagogy. Integrated Science shows 10% of prevalence, while Chemistry records the least with only 5%. Nevertheless, challenges such as limited resources, rigid curriculum, and inadequate teacher training on monitoring students’ projects and providing adequate assessment were also identified. This study recommends a need for teacher training and resources mobilization in schools support educators to effectively embrace Project-based learning pedagocy in mathematics and science subjects.

## Introduction

Conventional approaches to Mathematics and Science instruction are frequently criticized for overemphasizing rote memorization, offering minimal practical application, and failing to link learning to authentic contexts. However, the integration of project-based learning (PjBL) in Mathematics and Sciences (Biology, Chemistry, Physics, and Computer science) has received much attention because of its potential to engage students and expose them to real-world problem-solving ( Han et al., 2016). Project-based learning has gained popularity in educational research and curriculum changes as a viable teaching strategy for integrated science education, to improve students’ competencies needed for the 21st Century ( Serin, 2019). Project-based learning pedagogy attracts students’ attention and curiosity by letting them work on projects that apply to everyday situations whereby students face challenges to develop solutions to real-world problems and questions ( Chin, 2015). PjBL aligns with constructivist learning theories, which suggest that learners actively construct knowledge rather than passively receiving information. Instead, the content knowledge and skill development are significantly emphasized ( Haatainen & Aksela, 2021). Through project-based learning instructions, students are encouraged to learn independently to manage their tasks while performing activities to seek answers to the identified problems ( Serin, 2019).

Project-based learning-based education is intrinsically meaningful since it is grounded and involves mature competencies like creativity, critical thinking, collaboration, and communication. During Project-based learning, teachers mentor and counsel students ( Wurdinger & Rudolph, 2009). Project-based learning is known for its attributes to develop both technical and non-technical skills. For instance, through Project-based learning, students gain valuable life skills that instill confidence, and interest, and equip them with skills and desires to become self-directed lifelong learners in Mathematics and Sciences ( Wurdinger & Rudolph, 2009).

To implement project-based learning-based instruction, students in collaboration with teachers identify the potential problems in their environment. Students collaborate in groups to find solutions to complex issues grounded in the curriculum ( Han et al., 2016). Students choose what activities to engage in and how to tackle the challenge. Students collect data from many sources, synthesize it, examine it, and draw knowledge from it ( Haatainen & Aksela, 2021). This is a genuine inquiry involving students initiating the process with their questions, embarking on a quest for resources to test ideas and draw conclusions ( Larmer & Mergendoller, 2010). To this end, students discover greater significance in project work when they engage in genuine inquiry, rather than simply retrieving and copying information from books or websites.

Teachers employ various strategies to enhance student engagement and understanding. These strategies collectively aim to make learning Mathematics and Science subjects more engaging, relevant, and effective for diverse learners. These include inquiry-based learning, where students actively explore concepts through experiments and problem-solving, fostering critical thinking and curiosity ( Twahirwa et al., 2021). Collaborative learning is also widely used, encouraging students to work in groups to discuss and solve complex problems, and promoting peer learning and communication skills ( Han et al., 2016). Technology integration, such as simulations, interactive software, and digital tools, helps visualize abstract concepts and makes learning more dynamic ( Iyamuremye et al., 2022). Formative assessments, like quizzes and hands-on activities, are frequently utilized to provide immediate feedback, guiding students and teachers in addressing learning gaps ( Ukobizaba et al., 2021). However, there is a growing focus on Science and Mathematics education. There is a need for innovative teaching approaches that foster critical thinking, collaboration, and problem-solving skills ( Serin, 2019).

Project-based learning has emerged as a promising pedagogical method for engaging students in meaningful, real-world applications of Science and Mathematics concepts ( Wurdinger & Rudolph, 2009). Studeis were conducted on the contributions of project-based learning, but a focus was put on students’ subject performance. Aspects such as the contributions of PjBL on students’ engagement, creativity and communication, and conceptual understanding are not sufficiently explored ( Filcik et al., 2012). The reviewed studies were conducted on the benefits of Project-based learning but lacking a comprehensive synthesis of the contribution, prevalence, and challenges of PjBL pedagogy in Mathematics and Science education. This filling this gap yields the educators awareness, interest, and insights in applying this innovative pedagogy into their regular instructions. Indeed, traditional teaching still dominates mathematics and science instructions within primary and secondary schools in sub-saharan African countries. Teaching students to memorize concepts. A gap was found in teachers’ awareness about the contribution of PjBL in enhancing students conceptual understaaning and skills development. In addition, reasons behing teachers relying on traditional teaching and challenges associateed with the teachers’ implementation of PjBL are not examined. Thus, the contributions, prevalence, and challenges of PjBL in mathematics and science subjects should urgently be explored. The findings from this study provides with education stakeholders, curriculum developers and teachers with awareness about the effectivenss of PjBL in Mathematics and Science education. The study also consolidates and provides areas requiring more support in teaching and learning mathematics and science subjects. This review of literature sought to answer the following research questions:

(1) What are the contributions of project-based learning on students’ achievement in mathematics and science?
(2) To what extent do project-based learning pedagogy prevail in mathematics and science instruction?
(3) What challenges do teachers face in implementing Project-Based Learning in mathematics and science instruction?

## Methodology

A systematic review was used to collect and analyze data ( Pati & Lorusso, 2018). To collect data, keywords such as “project-based learning and students’ academic development”, “project-based learning and students’ engagement”, “project-based learning and students’ skills development”, “project-based learning and students’ performance in Mathematics and Sciences”, “project-based learning and challenges”, were used to access and download resources. Using search engines such as Google Scholar, Academia, Search 4 Life, Scopus, and Web of Science, 202 were downloaded. These databases were deemed valid, relevant, and reliable based on their high reputation as a repository of high-quality academic studies. However, inclusion and exclusion criteria followed. Within this regard, 27 duplicated papers were filtered out and 175 articles remained. Next, 115 papers not related to project-based learning were filtered out. A deep filtering was conducted. Thirty-seven (37) article papers that talked about PBL but did not directly reflect the study’s constructs were also filtered out. Therefore, 20 studies remained and were used for analysis. The downloaded papers were published from 2000 to 2023. This period was significant for collecting current and updated data about the contribution of Project-based learning in Mathematics and science education.

For transparency, completeness, and quality of the study, the risk of bias assessment was controlled for all employed literature by considering study design, sample selection, clarity of intervention implementation, outcome measurement. In this regard, we have examined whether the included studies selectively reported outcomes related to PjBL contributions, prevalence, and challenges. Therefore, evidence was evaluated qualitatively based on study design, methodological rigor, consistency of findings, and relevance to the review of literature research questions. A Preferred Reporting Items for Systematic Reviews and Meta-Analyses (PRISMA) check list ( Page et al., 2021) ensuring transparency, completeness, and quality of the study was also provided and found at Figshare repository ( Ukobizaba et al., 2025).

**Figure 1.**
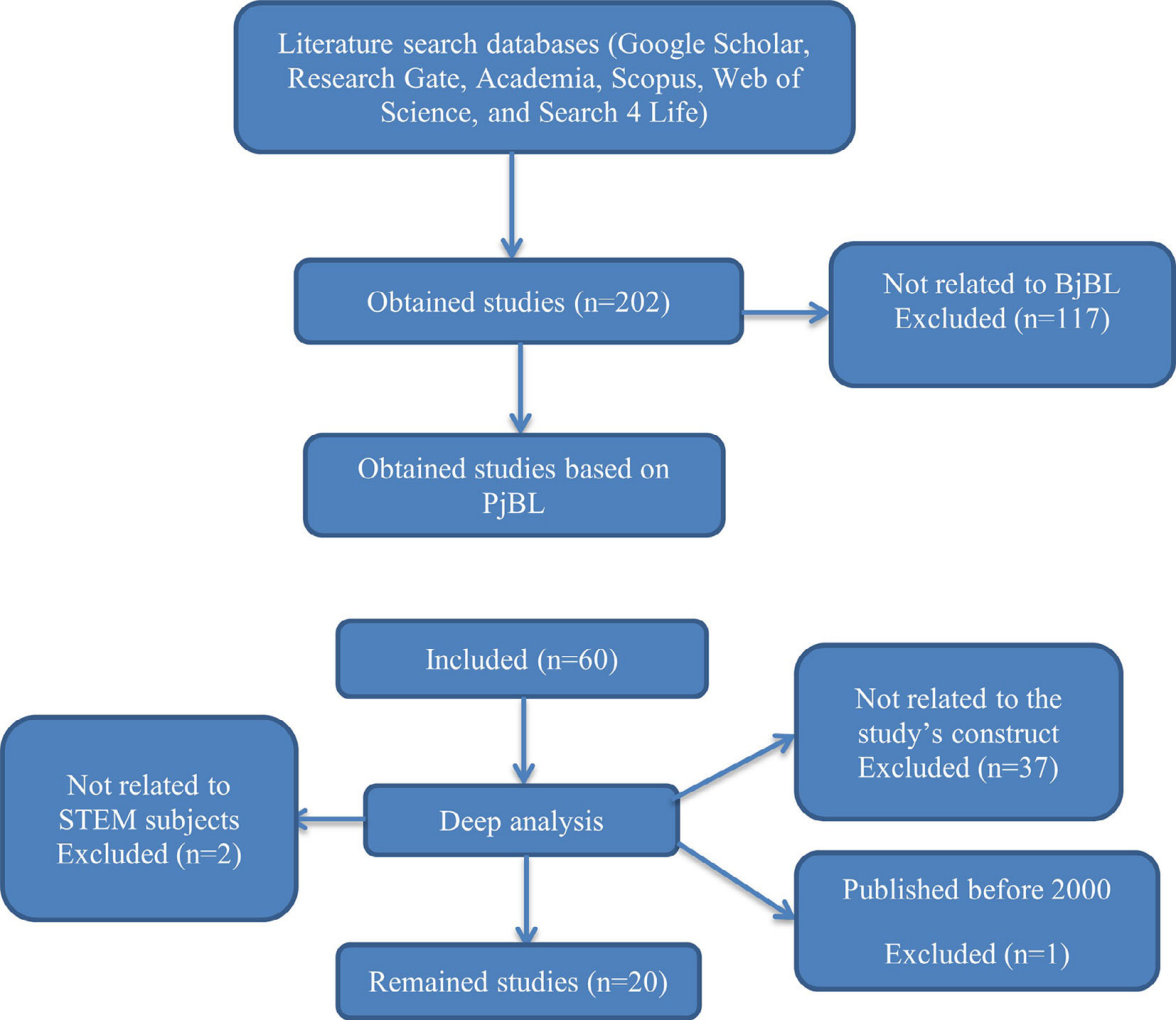
The PRISMA diagram of resources inclusion and exclusion process.

The review of literature was guided by the PRISMA framework, with clear inclusion and exclusion criteria ( Sarkis-Onofre et al., 2021). The Figure 1 was simplified for clarity.

## Results

Three of the reviewed literature was on the contribution of Project-based learning on students’ engagement. Eight of the reviewed literature were about the contribution of Project-based learning in enhancing students’ skills development. A significant part was occupied by the contribution of Project-based learning in enhancing students’ subject performance where 9 studies were reviewed. Three studies focused on the challenges linked to Project-based learning.

### i) The contributions of PjBL on students’ achievement in mathematics and science

**a) Enhancing students’ engagement**

Students’ engagement refers to the dynamic participation and involvement in different learning activities ( Larmer & Mergendoller, 2010). Three of the reviewed studies, confirmed that Project-based learning enhances students’ engagement (eg. Doppelt, 2003; Almulla, 2020; Gasana et al., 2023). For instance, in the study on the effectiveness of PjBL approach to engage students in learning, Almulla (2020) employed a questionnaire to collect data on 124 teachers using the Project-based learning approach. The results showed that the Project-based learning technique improves student engagement in sharing information and discussion. In addition, Gasana et al. (2023) employed a quasi-experimental and non-equivalent group design with a quantitative research approach. The participants were 78 senior students. It was found that students who were taught through Project-based learning were motivated to learn linear motion since they were interacting with robots. Further, Pan et al. (2021) employed a student evaluation of teaching (SET) to assess the project-based learning program. Within this regard, a SET instrument was used to measure the effectiveness of project-based learning. Qualitative data was collected about how students collaborate with their classmates on projects while solving real-world problems. The results showed that students were actively involved in the implementation of projects.

**b) Enhancing students’ creativity and communication**

Eight studies were reviewed under this construct. The reviewed literature revealed that Project-based learning effectively promotes skill development, particularly critical thinking, creativity, leadership skills, and problem-solving (eg. Abidin et al., 2020; Yamin et al., 2020; Djam’An et al., 2021). For instance, Djam’An et al. (2021) evaluated the use of the Project Learning model in improving students’ creativity in building their cities using mathematics (geometry, area, and volume). The study employed 34 primary students. The results from the study concluded that the PjBL model could improve students’ abilities in building a city through mathematics concepts. In addition, Abidin et al. (2020) investigated the effects of Project-Based Learning-Literacy in Improving Students’ Mathematical Reasoning Abilities in Elementary Schools. Using the experimental method, two sample groups consisting of a control and an experimental group were formed. During the project implementation, contextual material with literacy works was made and students were invited to carry out the process of thinking about mathematical contexts in daily life. As a result, it was found that there are differences in the student’s ability to reason mathematically for students who acquire learning PjBL-literacy compared to conventional learning.

While assessing the effect of project-based learning on leadership abilities and communication skills, Sirotiak & Sharma (2019) accepted methodologies for construction engineering and management education for undergraduate students who need to learn a combination of technical and non-technical competencies. The study used a pre-experimental pre-test/post-test design. During implementation, students were given responsibilities to lead the team. The results showed a statistically significant difference (p < .01) between the pre-and post-test, indicating an improvement in the student’s ability to set goals, communicate, identify, and organize activities. In addition, Wurdinger and Rudolph (2009) conducted a study on a different type of success: teaching important life skills through project-based learning. Surveys were used to collect data from students, teachers, and parents on their perception of the school in developing important life skills such as creativity, finding information, problem-solving, and learning how to learn. The results from data analysis exhibited the four highest-ranked skills including creativity (94%), finding information (92%), problem-solving (89%), and learning how to learn (89%). These results indicate that students gained skills that help them to succeed in college, and life in general through Project-based learning. It enhances one’s ability to succeed in college more than learning academic skills.

**c) Enhancing students’ conceptual understanding**

The construct of students’ subject performance has occupied a big part of the reviewed studies; nine studies have fallen under this construct. The reviewed studies were conducted on students’ performance in mathematics ( Kholid et al., 2022; Holmes and Hwang, 2016), in sciences (eg. Gomez-del Rio & Rodriguez, 2022; Çakici, 2013), integrated science subjects (eg. Han et al., 2016), Physics (eg. Gasana et al., 2023; Twahirwa et al., 2021), and computer engineering (eg. Najeeb and Memon, 2022). The reviewed studies showed that PjBL contributes to enhancing students’ academic performance (eg., Wurdinger et al., 2020; Kholid et al., 2022; Twahirwa et al., 2021) except for Holmes and Hwang (2016) who found that there is no statistical significant contribution.

Taking some examples, Çakici (2013) examined the effect of project-based learning activities on fifth-grade children’s science achievement and their attitudes toward science courses. The study employed 44 fifth-grade students at a public primary school in the Northwestern part of Turkey. A quasi-experimental study was used. Students were divided into two groups a control group (CG, n = 22) and an experimental group (EG, n = 22). The EG employed project-based practices, while the CG used traditional teaching practices. Students in the EG carried out three science projects for the science unit on ‘sound’. The findings from the study showed that children’s science achievement significantly improved through project-based activities. In addition, Twahirwa et al. (2021) studied the Effect of Project-Based Learning: Learners’ Conceptualization and Achievement in Science Education. Using a quasi-experimental design, a pre-and post- Physics Achievement Test was administered. By comparing the control and experimental group results, the p-value was less than 0.05 (p < 0.05). These results indicated a positive effect on students’ conceptual understanding of Physics. However, Holmes and Hwang (2016) used a mixed-method and a longitudinal study to investigate the effect of project-based learning (PBL) on secondary mathematics students’ academic skill development. The participants of the study were the eighth and ninth-grade students. The results showed no overall statistical difference between the two schools in mathematics conceptual understanding (p > .05). Mathematics content learning in the two environments did not seem to make a difference.

### ii) Prevalence of PjBL in mathematics and science

**Figure 2.**
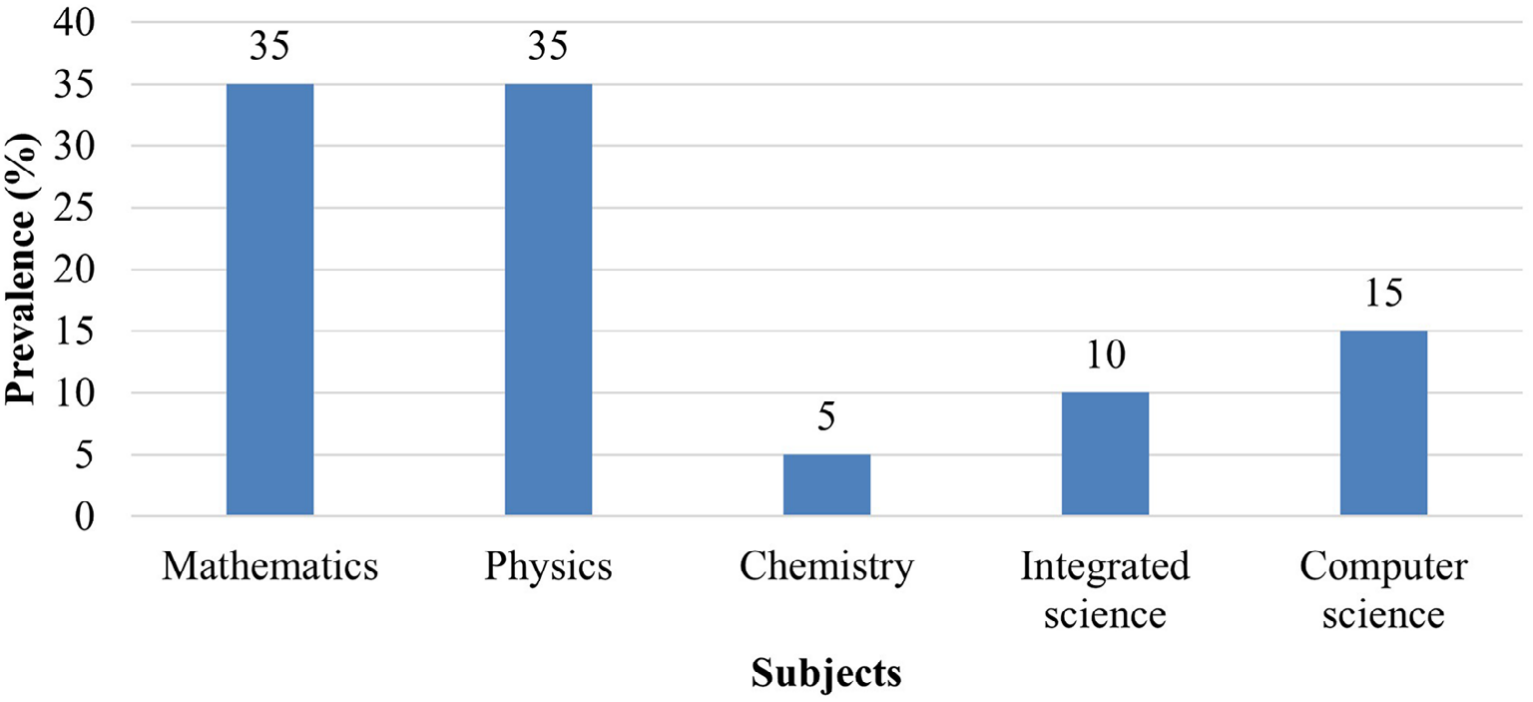
Prevalence of PjBL in Mathematics and science subjects.

Figure 2 shows the number of reviewed studies where project-based learning pedagogy wad applied in teaching Mathematics and science subjects. PjBL was used by teachers while Mathematics, integrated science, computer science, and physics. However, some reviewed literature showed that PjBL was conducted in other subjects. The majority of authors studied Mathematics and Science subjects. See Figure 2.

The data presents the prevalence of Project-Based Learning (PjBL) across mathematics and science subjects. Results indicated that PjBL prevalence was highest in mathematics and physics (35% each), while chemistry and biology accounted for 15% each. On the other hand, Computer Science accounts for 15%, Integrated Science shows 10%, while Chemistry records the least with only 5%. The higher prevalence of Project-Based Learning (PjBL) in Mathematics and Physics, each accounting for 35% of the reviewed literature, can be attributed to the inherently problem-solving and analytical nature of these subjects, which aligns well with the PjBL approach. Mathematics and Physics concepts often lend themselves to real-world applications, modeling, and hands-on experiments, making it easier for educators to design projects that engage students in inquiry, experimentation, and collaborative problem-solving. In contrast, Chemistry and Biology, which accounted for only 15% each, may present more logistical and safety challenges in implementing hands-on projects, such as the need for specialized laboratory equipment, controlled environments, and careful handling of chemicals or biological materials. Additionally, curriculum constraints and limited resources in many educational settings can make it more difficult for teachers to apply PjBL extensively in Chemistry and Biology, resulting in a lower representation in the literature. Overall, the alignment between subject characteristics, practical feasibility, and available resources likely explains the uneven distribution of PjBL prevalence across these STEM disciplines.

### iii) Challenges in Teachers’ incorporation of PjBL in mathematics and science

Three the reviewed studies reported the teachers’ challenges linked to PjBL incorporation into mathematics and science instruction. The challenges encountered by teachers included difficulties in selecting relevant content, time management, lack of abilities to monitor and provide adequate assessment, and lack of materials to implement students’ projects ( Aldabbus, 2018). During project implementation, teachers meet with difficulties in terms of time as project-based learning takes a long time, and others feared to disturb the learning process ( Trisdiono, 2014). For example, Shome and Natarajan (2013) reported that teachers felt too burdened with work for conducting projects within school hours and inadequate resources. The reviewed literature showed that teachers experienced difficulties in doing authentic assessments in the learning process and learning outcomes, especially in attitudes and skills ( Trisdiono, 2014).

## Discussion

The reviewed literature showed that PjBL has emerged as a transformative educational approach with significant contributions to various facets of student development. The purpose of employing different teaching and learning approaches and strategies is to make students successful in different learning subjects ( Han et al., 2016). The majority of the reviewed studies were on the contribution of Project-based learning on students’ subject performance (eg., Grant & Branch, 2005; Twahirwa et al., 2021) followed by Project-based learning in enhancing students’ skills such as the developments of communication, collaboration, creativity, and critical thinking ( Filcik et al., 2012; Kholid et al., 2022).

Project-based learning enhances students’ engagement by fostering active participation, collaboration, and interest through hands-on, real-world problem-solving activities. The current trend of developing 21 ^st^-century skills requires students to be involved in constructing their knowledge. Indeed, the application of Project-based learning goes hand in hand with the application of active learning pedagogy whereby students are actively engaged in solving real-world problems through authentic tasks. For instance, the reviewed studies were on applying Project-based learning to engage students in different subjects ( Almulla, 2020). Indeed, Filcik et al. (2012) and Pan et al.(2021) argued that students’ active engagement and interest have increased while learning science and mathematics. Thus, through Project-based learning, students will find the meaning and application of learning concepts acquired in class to real-life situations, which will in turn enhance their interest in learning those subjects.

The reviewed literature showed that Project-based learning supports skills development, equipping students with critical thinking, communication, teamwork, and self-management. BjBL was fought as one of the teaching approaches to enhance 21 ^st^-century skills ( Serin, 2019). Within the same vein, a competence-based curriculum currently used in different countries was conceived and developed to develop students skills such as leadership abilities skills ( Walters and Sirotiak, 2011), improving students’ Mathematical reasoning abilities ( Abidin et al., 2020) and teaching important life skills through project-based learning ( Wurdinger and Rudolph, 2009). We agree with Trisdiono (2014) who argued that teachers should teach and give authentic assessment based on students’ context to enhance their skills. If students have effectively acquired subjects’ concepts through Project-based learning transfer the acquired knowledge into their careers ( Wakumire et al., 2022). It is expected that teaching students through Project-based learning would increase the number of competent human resources ready to apply skills in their future careers ( Han et al., 2016). Project-based learning prepares students to solve real-life problems.

Based on the reviewed literature, Project-based learning positively impacts students’ subject performance. It enables students to understand the content. For instance, Han et al. (2016) and Twahirwa et al. (2021) observed higher performance in Sciences and Mathematics due to PjBL. Similarly, Abidin et al. (2020) found statistically significant improvements in mathematical reasoning and geometry scores. These findings highlight the potential of Project-based learning to improve academic outcomes, particularly when properly aligned with curricular goals and adequately supported. Though, Holmes and Hwang (2016) noted no significant academic differences in some cases. The traditional teaching strategy used to make Mathematics and Sciences abstract which was difficult for students to retain related concepts. However, when students receive authentic learning, as it is the key principle of Project-based learning pedagogy, students are likely to understand mathematics and science concepts ( Wurdinger & Rudolph, 2009). Therefore, Project-based learning is expected to enhance students’ conceptual understanding.

Teachers frequently face barriers such as inadequate training, scarce resources, and insufficient institutional support when attempting to apply PjBL effectively. Problems linked to Project-based learning should be taken into consideration and solutions such as encouraging students to complete their projects, optimizing the teacher’s role as supervisor, schools to finance the students’ projects, selecting projects suitable for the available resources, and facilitating teachers in their daily activities related to Project-based learning ( Cintang et al., 2018). Therefore, Project-based learning shouldn’t be viewed as a burden for teachers, but as an effective way to implement a student-centered approach ( Shome and Natarajan, 2013).

The findings highlight that PjBL is most widely applied in physics and mathematics, whereas chemistry and biology remain comparatively underexplored, possibly due to laboratory constraints and curricular structures. These results reflect the role of Mathematics and Physics across STEM disciplines and its alignment with Project-based learning’s emphasis on problem-solving and analytical skills ( Wakumire et al., 2022). In addition, Physics contributes in emphasizing its experimental nature and suitability for hands-on activities ( Çakici, 2013). These findings align with existing literature that recognizes Project-based learning as particularly effective in subjects requiring critical thinking and practical application ( Abidin et al. 2020). Therefore, more teachers should incorporate Project-based learning in different subjects, particularly Mathematics, and sciences to make these subjects more relevant to students.

Based on the reviewed literature, the pedagogical implications of PjBL are significant for teachers, policymakers, and curriculum developers. Teachers can leverage PjBL to actively engage students in meaningful learning experiences, fostering not only conceptual understanding in Mathematics and Science but also critical life skills such as creativity, problem-solving, communication, and leadership. Policymakers can support the integration of PjBL by providing adequate resources, training programs, and flexible timetables that allow for extended project implementation, addressing common challenges such as time constraints and lack of facilities. Curriculum developers can design learning modules that embed real-world, inquiry-based projects aligned with subject concepts, ensuring that students’ learning is both practical and cognitively stimulating. Overall, the findings underscore that implementing PjBL can enhance academic achievement while preparing students for collaborative and innovative participation in future academic and professional contexts.

## Conclusion

This review underscores the substantial benefits of PjBL in enhancing learning outcomes, while also acknowledging persistent challenges that must be addressed through targeted teacher training, curriculum flexibility, and resource allocation. The reviewed literature showed that Project-based learning enhanced students’ engagement, collaboration, creativity, and conceptual understanding of Mathematics and Sciences. The reviewed literature showed that PjBL dominates in Mathematics and Physics with the highest prevalence while Chemistry records the least prevalence. Although PjBL was found effective, it has associated challenges. These include teachers’ limited skills and inability to choose significant content, time management, monitoring, and assessment. These challenges underline the need for proper planning, teacher training, and institutional support to mitigate barriers and maximize the effectiveness of PjBL. The following review of the literature provides insights into further research. To this end, further research should not only address contributions, prevalence, and challenges, but also explore longitudinal impacts of PjBL on students’ lifelong learning and transferable skills.

## Declarations and statements

### Ethics and consent

No ethical approval or consent required.

## Reporting guidelines

Figshare: PRISMA checklist for [Project-Based Learning in Mathematics and Science: A Review of Contributions, Prevalence, and Challenges]. https://doi.org/10.6084/m9.figshare.30173854.v2 ( Ukobizaba et al., 2025).

Data are available under the terms of the Creative Commons Zero “No rights reserved” data waiver (CC0 1.0 Public domain dedication).

## Data Availability

No underlying data associated with this article. Figshare: [Project-Based Learning in Mathematics and Science: A Review of Contributions, Prevalence, and Challenges]. https://doi.org/10.6084/m9.figshare.30173854.v2 ( Ukobizaba et al., 2025). This project contains the following extended data:
○
Table 2_Reviewed literature.docx ○ Review protocol.docx Data are available under the terms of the Creative Commons 1.0 Universal License (CC0 1.0).
