# Project-based learning in STEAM subjects for socioformation: a systematic review of contributions, prevalence, and challenges

**DOI:** 10.12688/f1000research.170698.2

**Published:** 2026-03-31

**Authors:** Fidele Ukobizaba, Jean Francois Maniraho, Alphonse Uworwabayeho

**Affiliations:** 1 African Centre of Excellence for Innovative Teaching and Learning Mathematics and Science (ACEITLMS), University of Rwanda College of Education (URCE), Kayonza, P.O Box 55 Rwamagana, Rwanda

**Keywords:** Contributions of project-based learning, challenges, Mathematics and Science education, skills development, prevalence of project-based learning

## Abstract

While traditional Project-based Learning (PjBL) enhances engagement, creativity, and conceptual understanding, emerging socioformative approaches are pedagogies that highlight the need for interdisciplinary, ethically grounded projects addressing real-world challenges. This study employed a systematic review to investigate the contributions, prevalence, and challenges of PjBL in Science Technology, Engineering, Arts, and Mathematics (STEAM) education while enhancing socio-critical competencies. Thus, 211 articles were downloaded from Google Scholar, Academia, Search 4 Life, Scopus, and Web of Science databases. Through the rigorous filtering processes, 32 articles fell into the study’s scope and were considered and used for analysis. The results from the reviewed studies showed that project-based learning contributes to enhancing students’ engagement, skills development, and conceptual understanding in STEAM subjects. Most of the reviewed studies employed traditional PjBL, while a few studies’ methodologies considered trending projects involving transferable skills and socioformation development. An even distribution of PjBL prevalence was found across STEAM subjects, with the highest prevalence in mathematics and science. Also, the reviewed studies marked the highest prevalence of PjBL in higher education and high school education. Studies on PjBL are dispersed across different countries, with the highest prevalence in Indonesia and Spain. Nevertheless, lack of support, challenges such as limited resources, rigid curriculum, teachers’ inability to select relevant content, and inadequate teacher training on monitoring students’ projects and providing adequate assessment were also identified. The review provided recommendations, including future directions for research, particularly in relation to education for sustainable social development and AI-supported learning environments.

## Introduction

Conventional approaches to Science Technology, Engineering, Arts, and Mathematics (STEAM) instruction are frequently criticized for overemphasizing rote memorization, offering minimal practical application, and failing to link learning to authentic contexts. However, the integration of project-based learning (PjBL) in STEAM subjects has received much attention because of its potential to engage students and expose them to real-world problem-solving ( Han et al., 2016). Project-based learning has gained popularity in educational research and curriculum changes as a viable teaching strategy for integrated science education, to improve students’ competencies needed for the 21st Century ( Serin, 2019). Project-based learning pedagogy attracts students’ attention and curiosity by letting them work on projects that apply to everyday situations, whereby students face challenges to develop solutions to real-world problems and questions ( Almulla, 2020).

In addressing contemporary educational challenges, this review adopts a constructivist framework emphasizing learning objectives ( Haatainen & Aksela, 2021) and expands to incorporate more flexible, student-centered approaches that emphasize critical and socio-critical thinking known as the socioformative approach ( Flores & Esteva, 2025). This approach aims to prepare students not only intellectually but also socially, equipping them to engage responsibly with complex societal issues in a rapidly evolving and technology-driven world ( Wan, 2025). Through this approach, students develop a sense of social responsibility, ethical decision-making, and preparation for the future ( del Carmen López-Quesada et al., 2022). Project-based learning is known for its attributes to develop both technical and non-technical skills. Project-based learning-based education is intrinsically meaningful since it is grounded and involves mature competencies like creativity, critical thinking, collaboration, and communication. Through the socioformation approach, the teacher’s role shifts significantly from being a traditional knowledge transmitter to a guider, facilitator, and mentor while supporting both the cognitive and social development of students ( Morcom & MacCallum, 2022). Thus, Project-based learning is sought to equip students with valuable life skills that instill confidence and interest, and equip them with skills and desires to become self-directed lifelong learners ( Wurdinger & Rudolph, 2009).

To implement project-based learning-based instruction, students, in collaboration with teachers, identify the potential problems in their environment. Students collaborate in groups to find solutions within the environment to complex issues grounded in the curriculum ( Han et al., 2016). Students choose what activities to engage in and how to tackle the challenge. Students collect data from many sources, synthesize it, examine it, and draw knowledge from it ( Haatainen & Aksela, 2021). This is a genuine inquiry involving students initiating the process with their questions, embarking on a quest for resources to test ideas and draw conclusions ( Larmer & Mergendoller, 2010). To this end, students discover greater significance in project work when they engage in genuine inquiry, rather than simply retrieving and copying information from books or websites.

Teachers employ various strategies to enhance student engagement and understanding of different learning subjects. These strategies collectively aim to make learning subjects more engaging, relevant, and effective for diverse students. These include inquiry-based learning, where students actively explore concepts through experiments and problem-solving, fostering critical thinking and curiosity in collaboration with peers ( Twahirwa et al., 2021). Collaborative learning is widely used, encouraging students to work in groups to discuss and solve complex problems, and promoting peer learning, communication skills, and ethics ( Han et al., 2016). Technology integration, such as artificial intelligence (AI), simulations, interactive software, making plans, searching for content, helps visualize abstract concepts to make PjBL more dynamic. In addition, formative assessments, like quizzes and hands-on activities, are frequently utilized to provide immediate feedback, guiding students and teachers in addressing learning gaps ( Ukobizaba et al., 2021). Similarly, formative projects are used to track students’ learning improvement. Within this regard, modified PjBL approaches such as socioformation, combined with the integration of technologies like AI, are essential for learning different subjects because they foster critical thinking, interdisciplinary understanding, social responsibility, and active student engagement in real-world, technology-driven contexts.

Project-based learning has emerged as a promising pedagogical method for engaging students in meaningful, real-world applications of mathematics and science concepts ( Wurdinger & Rudolph, 2009). Studies were conducted on the contributions of project-based learning to enhance students’ subject performance (e.g., Holmes & Hwang, 2016; Najeeb & Memon, 2022; Gomez-del Rio & Rodriguez, 2022; Yamin et al., 2020). However, aspects such as the contributions of PjBL students’ engagement, creativity, and communication, and conceptual understanding for socioformation, combined with the integration of technologies like AI, are not sufficiently explored. In addition, mathematics and science instruction within sub-Saharan African countries is still dominated by students memorizing concepts. Project-based learning (PBL) has been widely recognized for its contributions to student achievement in mathematics and science. Yet traditional PjBL approaches often remain disciplinary and content-focused, emphasizing learning objectives over real-world or community-based problems. These limitations can reduce its effectiveness in addressing interdisciplinary challenges and preparing students for the rapidly evolving, AI-driven educational landscape. Emerging methodologies, such as socioformative projects and STEAM-based projects, offer more socially and technologically relevant approaches by integrating ethical, collaborative, and problem-solving competencies. By filling this gap, this review of literature yields the educators’ awareness and purposeful application of this innovative pedagogy to their regular instruction. This review examines the contributions, prevalence, and challenges of PBL implementation in STEAM subjects, highlighting opportunities to align PjBL with contemporary pedagogical demands such as student-centered learning, critical and creative thinking, interdisciplinary learning, digital and technological literacy, collaboration, and ethical and socio-critical awareness.

This review of literature sought to answer the following research questions:

1) To what extent does PjBL contribute to students’ engagement, skills, and subject performance for socioformation?
2) To what extent does Project-Based Learning prevail across different STEAM subjects, education levels, and countries?
3) What challenges are linked to the implementation of Project-Based Learning in STEAM education?

## Methodology

A systematic review was used to collect data. A systematic keyword combination made using Boolean operators (AND, OR, NOT) was used ( Dinet et al., 2004). Thus, keywords such as “project-based learning”, “project-based learning and students’ engagement”, “students’ skills development”, “students’ performance in mathematics and sciences”, “challenges”, socioformative projects,” “formative projects,” “STEAM education,” “AI in PBL,” “sustainability competencies,” “socioformation,“ and “sustainable social development,” were used to access and download resources. Using search engines such as Google Scholar, Academia, Search 4 Life, Scopus, and Web of Science, 211 resources were downloaded. These databases were deemed valid, relevant, and reliable based on their high reputation for holding high-quality academic studies.

The downloaded resources were analyzed to decide which materials are to be included and excluded before analysis. At first, duplicates were removed. Hence, 28 duplicated papers were filtered out, and 183 articles remained. In addition, 116 resources were filtered out because they related to project-based learning but for other subjects rather than in STEAM subjects. In addition, reviews of literature articles were also filtered out. Further, articles presented in conference proceedings were removed because they usually have limited peer review, preliminary data, or incomplete reporting, which can affect the reliability and quality of evidence. Thus, 67 remained. Furthermore, to get eligible studies for analysis, a deep screening was conducted to filter out studies focused on PjBL in non-STEAM disciplines. In this regard, studies were excluded if they (i) addressed general pedagogy without a clear PjBL intervention, (ii) lacked empirical data on contributions, prevalence, or implementation challenges, (iii) were purely theoretical or opinion pieces, or (iv) did not provide sufficient methodological detail to judge study quality. Therefore, 32 studies remained and were used for analysis. The downloaded papers were published from 2015 to 2026. This publication range was considered to get findings that are more relevant to the current educational context. Through studies screening, a narrative synthesis approach, and statistical descriptive analysis were used.

To ensure consistency in study selection, two independent reviewers screened all titles, abstracts, and full-text articles according to the predefined inclusion and exclusion criteria. Inter-rater reliability was assessed using Cohen’s kappa, which indicated substantial agreement (κ = 0.78). Any disagreements were resolved through discussion until a consensus was reached. In addition, transparency, completeness, and quality of the study, and the risk of bias assessment were controlled for all employed literature by considering study design, sample selection, clarity of intervention implementation, and outcome measurement.

**Figure 1.**
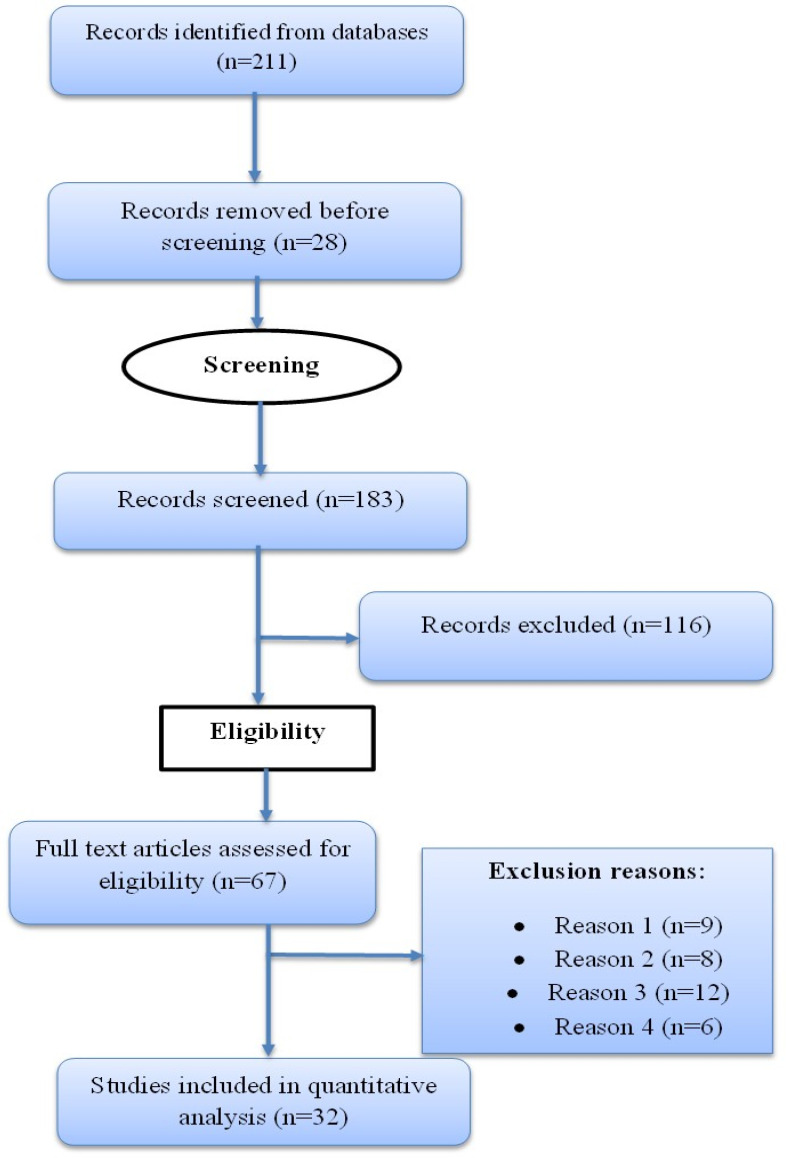
The PRISMA diagram for sources inclusion and exclusion process.

The review of literature was guided by the PRISMA framework, with clear inclusion and exclusion criteria ( Sarkis-Onofre et al., 2021). Figure 1 was simplified for clarity as shown below.

## Results

The results are drawn from 32 reviewed studies. To analyze data, results were categorized depending on reported multiple learning outcomes, such as engagement, skills, or performance, and challenges to avoid double-counting. In addition, a systematic review synthesizes the prevalence of PjBL based on the distribution of studies in STEAM subjects, education level, and the frequencies of conducted studies per country.

### i) The contributions of PjBL in STEAM subjects

The reviewed studies under the contribution were categories in three aspects including engagement, skills, and performance. The engagement category includes studies where the primary outcome was students’ level of participation, motivation, interest, or involvement in learning activities. The students’ engagement involves active learning for socioformation. Socioformation as a paradigm of innovation and educational experience aims to identify and solve problems that transform the social-educational-community environment through collaboration, interdisciplinarity, multidisciplinarity, innovation, and systematized projects integrated into education ( Flores & Esteva, 2025). The skills aspect is indicated by studies where the main outcomes were transferable competencies, not subject scores. The performance category includes studies where the main outcome variable was academic achievement or conceptual mastery.

***a) Enhancing students’ engagement for socioformation***

Students’ engagement refers to the dynamic participation and involvement in different learning activities ( Larmer & Mergendoller, 2010). The reviewed studies confirmed that Project-based learning enhances students’ engagement (e.g., Almulla, 2020; Gasana et al., 2023). For instance, in the study on the effectiveness of PjBL approach to engage students in learning, Almulla (2020) investigated the Effectiveness of the Project-Based Learning (PBL) Approach as a Way to Engage Students in Learning. To carry out a study, questionnaires were employed to collect data on 124 teachers using the project-based learning approach. The results showed that the learning projects enhanced student engagement in sharing information and discussion. In addition, Gasana et al. (2023) employed a quasi-experimental and non-equivalent group design with a quantitative research approach. There were 78 senior students. It was found that students who were taught through project-based learning were motivated to learn linear motion while interacting with robots. Further, Pan et al. (2021) employed a student evaluation of teaching (SET) to assess the project-based learning program. Within this regard, a SET instrument was used to measure the effectiveness of project-based learning. Qualitative data was collected about how students collaborate with their classmates on projects while solving real-world problems. The results showed that students were actively involved in the implementation of projects.

While the new trend of PjBL intends to develop socioformation, most of the reviewed literature, PBL methodologies employed focused on achieving learning processes (constructivist approach), but did not truly address territorial issues. The applied methodologies treat problems as a mere means for conceptual understanding (constructivism) and do not consider PjBL as a tool for socio-economic bond and environmental sustainability. Only two of the reviewed studies stressed developing social development. For instance, Farida and Rasyid (2019) conducted a study on The Effectiveness of Project-based Learning Approach to the social development of Early Childhood. The study employed a post-test only control design. The two random classes were selected, and each class consisted of 33 students. The control group continued their common learning activity while another group used a project-based learning approach. It was found that the project-based learning approach is effective in stimulating students’ cognition and social development in early childhood education. In addition, Holmes and Hwang (2016) explored the effects of project-based learning in secondary mathematics education. A mixed-method and longitudinal study was employed to investigate the benefits of project-based learning (PjBL) on secondary-mathematics students’ academic skill development and cognitive, social, and motivational learning outcomes. The findings suggested that PjBL students were more intrinsically motivated to solve problems in a social learning community. In addition, while assessing the effect of project-based learning on leadership abilities and communication skills, Sirotiak and Sharma (2019) recommended methodologies for construction engineering and management education for undergraduate students who need to learn a combination of technical and non-technical competencies. The study used a pre-experimental pre-test/post-test design. During implementation, students were given responsibilities to lead the team. The results showed a statistically significant difference (p < .01) between the pre-and post-test, indicating an improvement in the student’s ability to set goals, communicate effectively, identify, and organize learning activities.

***b) Enhancing students’ creativity for socioformation***

The reviewed literature revealed that project-based learning effectively promotes skill development, particularly critical thinking, creativity, leadership skills, and problem-solving (e.g., Abidin et al., 2020; Djam’An et al., 2021; Wurdinger et al., 2020; Yamin et al., 2020). For instance, Djam’An et al. (2021) evaluated the use of the project learning model in improving students’ creativity in building their cities using mathematics (geometry, area, and volume). The study employed 34 primary students. The results from the study concluded that the PjBL improves students’ abilities in building a city through the application of mathematics concepts. In addition, Abidin et al. (2020) conducted a study on the Effects of Project-Based Learning-Literacy in Improving Students’ Mathematical Reasoning Abilities in Elementary Schools. Using the experimental method, two sample groups consisting of a control and an experimental group were formed.

During the project implementation, contextual material with literacy works was made, and students were invited to carry out the process of thinking about mathematical contexts in daily life. It was found that there are differences in the students’ ability to reason mathematically for students who acquire PjBL-literacy compared to conventional learning.

In their study, Birdman et al. (2022) conducted a study on developing key competencies in sustainability through project-based learning in graduate sustainability programs. During the study, a two-year comparative case study follows the project-based-learning journeys of nine graduate sustainability students from three programs that were conducted for the Master of Sustainability at Arizona State University. It was found that defining aspects of project-based learning, including collaboration, student autonomy, and real-world connection, do contribute to students’ self-perceived competence development. In addition, Elvianasti et al. (2024) investigated the Effectiveness of Project-Based Learning on STEAM-Based student’s worksheet analysis with the Ecoprint Technique. The investigation was conducted as a quasi-experiment involving a sample size of 150 students selected through cluster sampling. Data collection was executed using standardized tests. The findings revealed that the implementation of project-based learning coupled with STEAM-based student worksheet analysis utilizing the ecoprint technique yields a substantial enhancement in learning motivation and student creativity. However, among the reviewed studies, none explicitly employed methodologies with the intention to solve the problem within a territory or leading to the context from the perspective of socioformation.

***c) Academic performance for environment transformation***

Project-based learning is applied to enhance students’ performance and environment transformation. However, most reviewed studies showed students’ performance in STEAM subjects (e.g., Chhabra & Gawande, 2023; Holmes & Hwang, 2016; Kholid et al., 2022; Gomez-del Rio & Rodriguez, 2022; Najeeb & Memon, 2022) with only one emphasizing environment sustainability ( Kricsfalusy et al., 2018).

For instance, Tika and Agustiana (2021) examined The Effect of a Blended Learning Project Based Learning Model on Scientific Attitudes and Science Learning Outcomes. The research sample amounted to 61 students sampling using the random sampling technique. The design uses Post-test Only Control Group Design. Data was analyzed through descriptive statistics and the SPSS-17 MANOVA test. There are differences in scientific attitudes and science learning outcomes. A significance level of 0.05 resulted in a p < 0.05. Similarly, Granado-Alcón et al. (2020) conducted a study on Project-Based Learning and the Acquisition of Competencies and Knowledge Transfer in Higher Education. The sample consisted of 464 students from the Universities of Huelva (N = 347; 74.8%) and Murcia (N = 117; 25.2%) in Spain, enrolled in the second year of a degree in either Infant or Primary Education. Data was collected through a self-administered questionnaire. The results showed that competencies were moderately acquired after PjBL. The findings revealed that students exhibited notable knowledge as well as a positive assessment of the project. Further, Demir and Önal (2021) conducted a study on the effect of technology-assisted and project-based learning approaches on students’ attitudes towards mathematics and their academic achievement. A quasi-experimental research design was used with pre-test, post-test. The findings of the research revealed that the PjBL approach had a higher level of effect on academic achievement. Furthermore, Han et al. (2016) investigated The Effect of Science, Technology, Engineering and Mathematic (STEM) Project Based Learning (PBL) on Students’ Achievement in Four Mathematics Topics. The longitudinal study was employed to compare the achievement of two groups of students who participated in the study in 2008, 2009, and 2010. The results showed that lessons integrating Science, Technology, Engineering, and Mathematics project-based learning improved students’ scores in mathematics in general (d = 1.311), algebra (d = 1.500), geometry (d = 1.837), and probability (d = .487), but not in problem solving (d = .343).

Most of the reviewed studies employed methodologies emphasizing students’ academic performance ignoring the role of PjBL on environment sustainability. Only one study was on environment sustainability. For instance, Kricsfalusy et al. (2018) conducted a study on Integrating problem- and project-based learning opportunities: assessing outcomes of a field course in environment and sustainability. A course developed for a professional master’s program in environment and sustainability that employs PjBL model. It was found that PjBL yields benefits such as strengthened environment sustainability, professional skills for students, and longitudinal research opportunities for teaching faculty.

PjBL generates activities and products that are automated in the AI era. However, few of the reviewed studies involved automated activities that can be generated by AI. For instance, Santos et al. (2026), conducted a study on Generative Artificial Intelligence (GAI) and Project-Based Learning Outputs in Technology-Enhanced Mathematics Education. A qualitative thematic analysis was used. The results showed that GAI with computational and dynamic geometry tools provides patterns of mathematical correctness, justifying classroom-oriented task types in mathematics. In the same vein, Ruiz Viruel et al. (2025) explored The Role of Artificial Intelligence in Project-Based Learning: Teacher Perceptions and Pedagogical Implications. A sample of teachers (n = 300) was involved in the study. It was found that AI-enhanced PjBL is rated significantly higher than regular PBL without AI. In addition, Wan (2025) conducted a study on the Impact of AI-Assisted Project-Based Learning Design on Innovation Ability. A quasi-experimental design was employed to apply AI-empowered PBL to the experimental group and traditional PBL to the control group. The results from the study revealed that the AI-Assisted group had a greater increase in problem-solving, critical thinking, and creative thinking abilities, with statistically significant differences.

While the AI-aided projects involve automatable products, most of the reviewed studies with different research designs, including exploratory, quasi-experimental, and longitudinal study (e.g., Lazić et al., 2021), which involve non-automatable processes. The reviewed studies involve aspects such as critical and socio-critical thinking, ethics, and awareness of sustainable social development ( Granado-Alcón et al., 2020; Luque-González et al., 2025). These results highlight the development of critical and socio-critical thinking, ethical awareness, and commitment to sustainable social development.

### ii) Projects-based learning prevalance

***a) Prevalence of PjBL in STEAM subjects***

**Figure 2.**
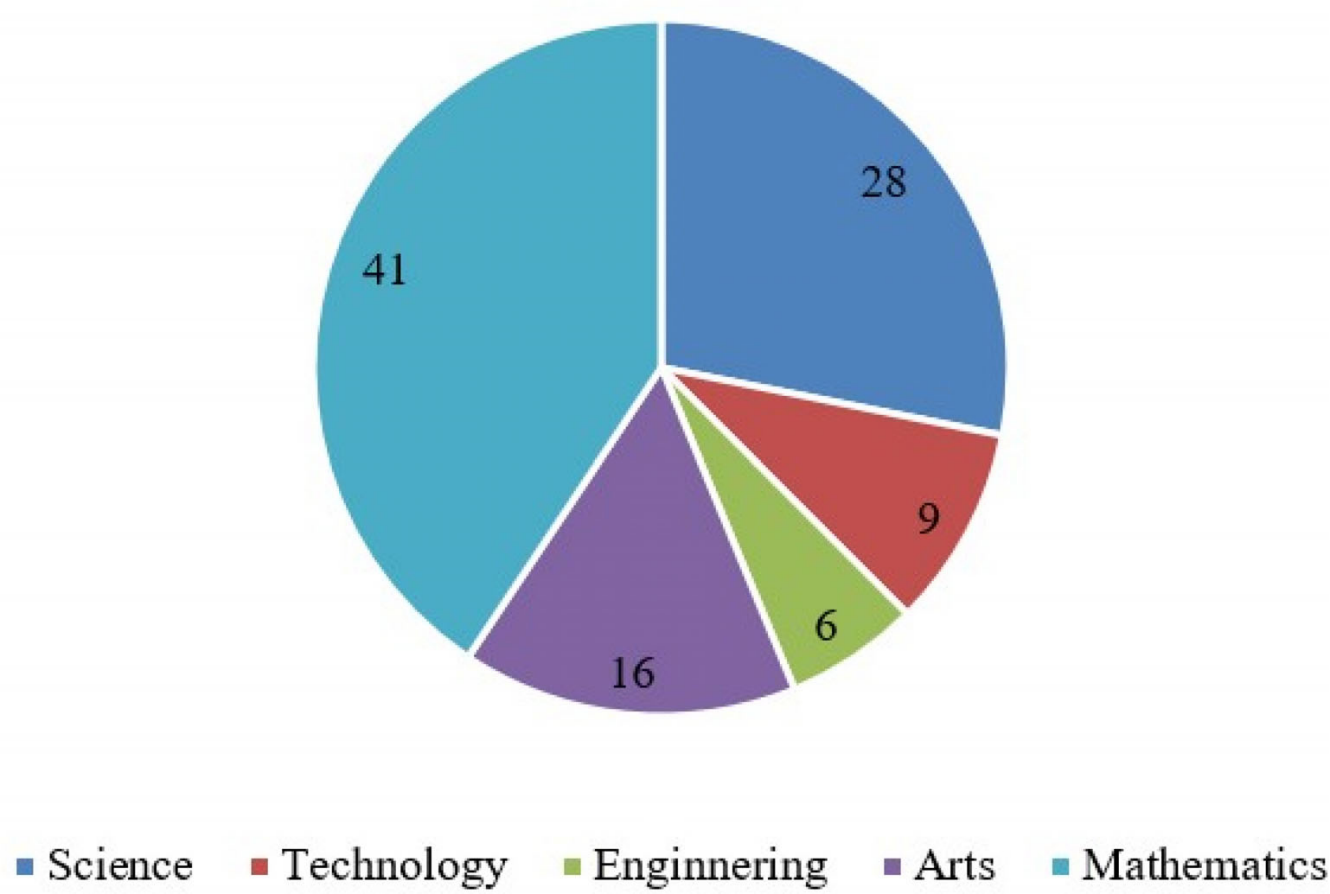
Prevalence of PjBL in STEAM subjects.

Figure 2 shows the extent to which Project-Based Learning prevails across different STEAM subjects. The prevalence in percentages was calculated based on 32 reviewed studies. See Figure 2.

Figure 2 represents the reviewed studies’ prevalence of PjBL across STEAM subjects. Results indicated that PjBL prevalence was highest in sciences (41%), followed by mathematics (28%). The low prevalence was found in Arts, Technology, and Engineering with 16%, 9%, and 6% of prevalence, respectively. Mathematics and science prevail since the concepts often lend themselves to real-world applications, modeling, and hands-on experiments, making it easier for educators to design projects that engage students in inquiry, experimentation, and collaborative problem-solving. In contrast, arts, technology, and engineering subjects may require more logistical and safety challenges in implementing hands-on projects, such as the need for specialized laboratory equipment and environment-related issues. Overall, the alignment between subject characteristics, practical feasibility, and available resources likely explains the uneven distribution of PjBL prevalence across these STEAM disciplines.

***b) Prevalence of PjBL per educational level***

**Figure 3.**
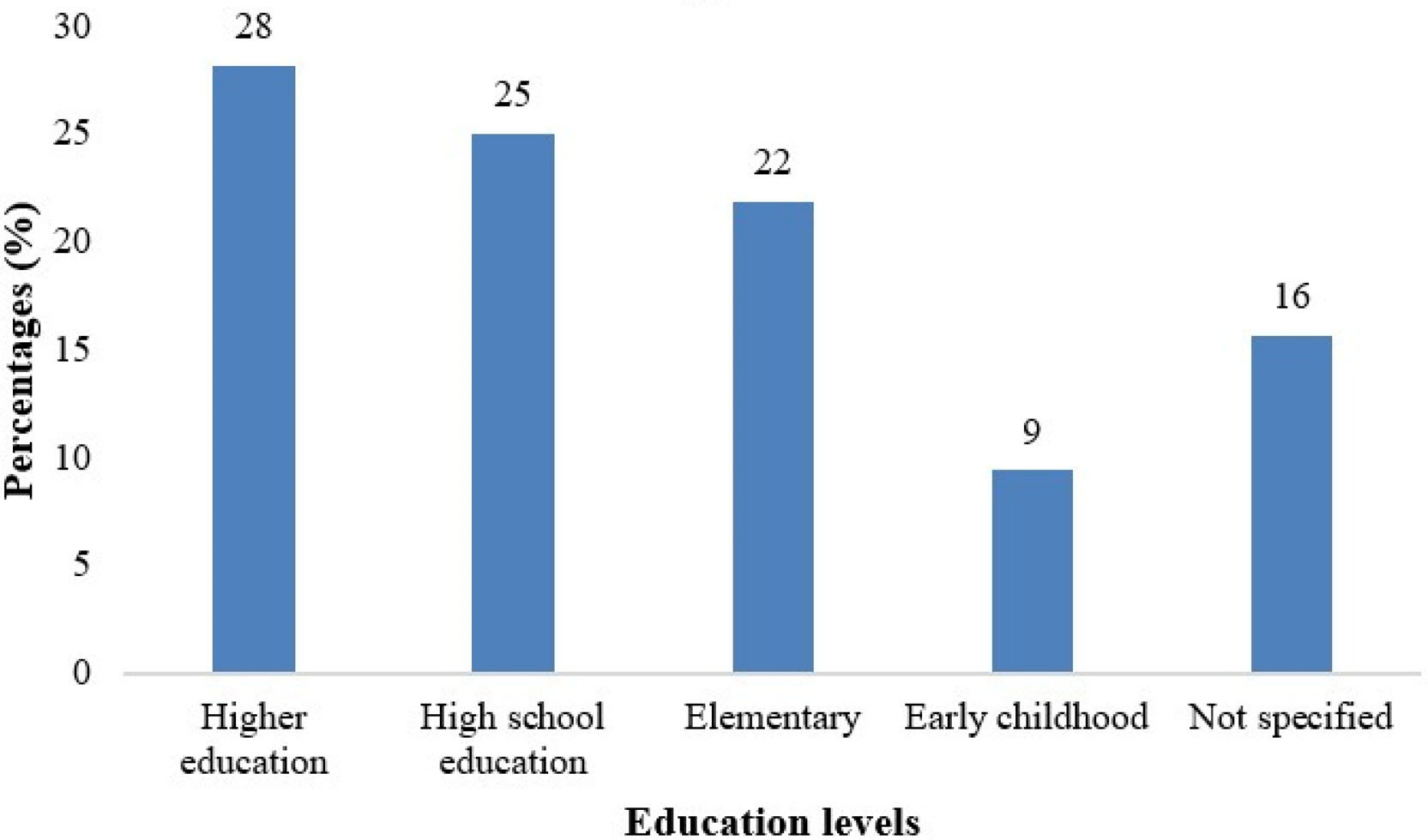
Reviewed studies by educational level.

The reviewed studies showed that PjBL prevails over early childhood education level through higher learning. Percentages were calculated based on 32 reviewed studies to show the extent to which PjBL prevails in each educational level. See Figure 3.

The reviewed literature showed that PjBL is applied throughout higher learning. However, the highest prevalence was found in higher education and decreased while moving to lower levels of education. For instance, in higher education, it is 28%, followed by 25% in high school education, while it is 22% and 9% in elementary and early childhood education, respectively. For 16% of studies, the level of education was not specified. The distribution indicates that PjBL research is represented across all educational levels, with high interest in higher learning institutions and in high schools, suggesting attention and engagement in elementary and early childhood education.

***c) Prevalence of PjBL in STEAM subjects per country***

**Figure 4.**
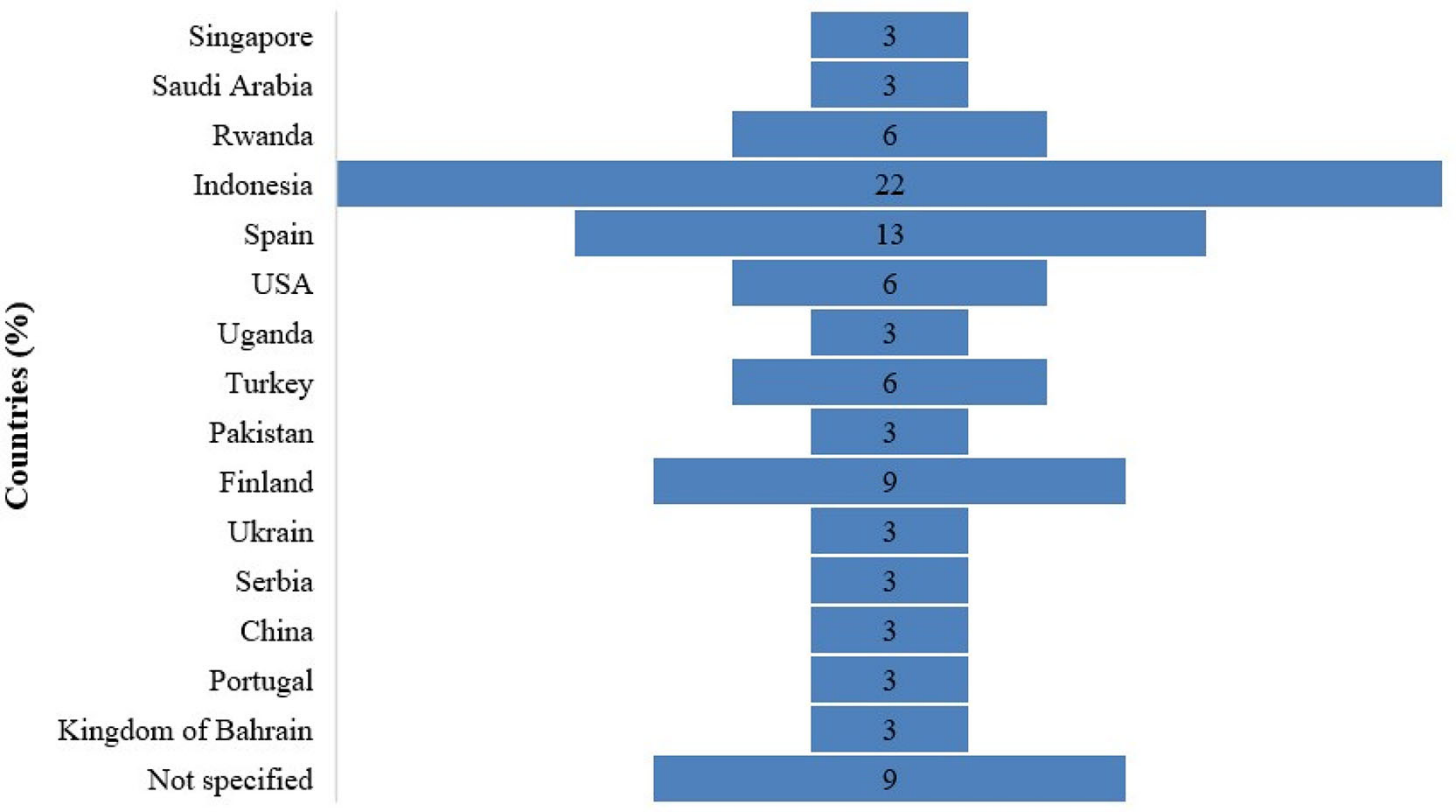
Prevalence of PjBL in STEAM subjects per country.

Figure 4 represents 15 countries in which studies were conducted in different countries across different continents. The percentages were calculated based on the 32 studies for each country. The percentages indicate the frequency with which a country appears in all reviewed studies. See Figure 4.

Results in Figure 4 show that studies were dominated in Indonesia (22%), Spain (13%), and Finland (9%). A medium proportion comes from countries such as Rwanda, the USA, and Turkey, with 6% each. The small portion was found for countries such as Singapore, Saudi Arabia, Uganda, Pakistan, Ukraine, Serbia, China, Portugal, Kingdom of Bahrain with 3% each. The relatively even distribution of PjBL prevalence across the countries suggests that PjBL has gained global pedagogical relevance due to its alignment with 21st-century competencies such as collaboration, problem-solving, and learner-centered instruction. However, the relatively modest percentages per country imply that the evidence base is dispersed rather than deeply concentrated within specific national contexts. However, the results limit the strength of context-specific conclusions or generalizations, given the limited sample of the reviewed studies.

### iii) Challenges linked to the incorporation of PjBL in STEAM education

The challenges encountered by teachers included difficulties in selecting relevant content, time management, lack of ability to monitor and provide adequate assessment, and a lack of materials to implement students’ projects. For instance, Viro et al. (2020) studied teachers’ perspectives on project-based learning in mathematics and science. The study was to investigate how Finnish pre- and in-service class teachers and mathematics and science teachers (N = 257) view PjBL. The results showed that PBL is suitable for both revising and learning new content. However, the relative inadequacy of resources and support for PjBL implementation was reported among challenges. In addition, Haatainem and Aksela (2021) carried out a study on Project-based learning in integrated science education: Active teachers’ perceptions and practices. The study employed a qualitative-led survey and a case study. 244 teachers from early childhood education to upper secondary school from 28 countries participated in the study. Among the results, challenges such as facilitating PjBL, such as time management or laborious planning, technical issues related to the use of different technological tools, and students who got tired because of heavy schedules, were reported.

## Discussion

While constructivist PjBL prioritizes knowledge construction, socioformative projects focus on community service, ethical engagement, and sustainable problem-solving, providing a pedagogical buffer against uncritical reliance on AI-generated solutions. The reviewed literature showed that PjBL has emerged as a transformative approach with significant contributions to various facets of student development. The purpose of employing different teaching and learning approaches and strategies is to make students successful in different learning subjects ( Han et al., 2016). The majority of the reviewed studies were conducted on the contribution of PjBL to students’ subject performance (e.g., Twahirwa et al., 2021), followed by students’ skills development, such as communication, collaboration, creativity, and critical thinking subjects ( Kholid et al., 2022).

Project-based learning enhances students’ engagement by fostering active participation, collaboration, and interest through hands-on, real-world problem-solving activities. The current trend of developing 21 ^st^-century skills requires students to be involved in constructing their knowledge. The application of project-based learning goes hand in hand with the application of active learning pedagogy, whereby students are actively engaged in solving real-world problems through authentic tasks. For instance, Pan et al. (2021) found that PjBL contributed to enhancing students’ active engagement and interest while learning science and mathematics. Thus, through project-based learning, students will find the meaning and application of learning concepts acquired in class to real-life situations, which will, in turn, enhance their interest in learning those subjects.

The reviewed literature showed that project-based learning supports skills development, equipping students with critical thinking, communication, teamwork, and self-management. PjBL was sought as one of the teaching approaches to enhance 21 ^st^-century skills ( Serin, 2019; Wurdinger et al., 2020). Within the same vein, through project-based learning, a competence-based curriculum currently used in different countries was conceived and used to develop students’ skills such as leadership abilities, improving students’ mathematical reasoning abilities ( Abidin et al., 2020), and life skills ( Wurdinger & Rudolph, 2009). Teachers should teach and give authentic assessments based on students’ context to enhance their skills. We concur with Wakumire et al. (2022) argued that if students have effectively acquired subject concepts, the acquired knowledge should be transferred and used in their careers. It is expected that teaching students through project-based learning would increase the number of competent human resources ready to apply skills in their future careers.

Based on the reviewed literature, project-based learning positively impacts students’ subject performance. Project-based learning enables students to understand the content. For instance, Han et al. (2016) and Twahirwa et al. (2021) observed higher performance in mathematics and science through PjBL. Similarly, Abidin et al. (2020) found statistically significant improvements in mathematical reasoning and geometry scores when PjBL was incorporated in instructions. These findings highlight the potential of project-based learning to improve academic outcomes, particularly when aligned with the curriculum. The effectiveness of PjBL can depend heavily on how well projects are aligned with tested content, how consistently the approach is implemented, and how familiar students and teachers are with inquiry-based methods. STEAM subjects’ concepts can be abstract, and assessments often emphasize procedural knowledge. Projects that are not tightly connected to curriculum goals or exam formats may not translate into measurable test-score gains, even if they support other outcomes like engagement or problem-solving skills. Contextual factors such as time constraints, teacher expertise, and school culture can also moderate PjBL’s academic impact. The traditional teaching strategy is used to make mathematics and science subjects more abstract. However, when students receive authentic learning, as it is the key principle of project-based learning pedagogy, students are likely to understand mathematics and science concepts ( Wurdinger & Rudolph, 2009). Therefore, project-based learning instructions should be employed to enhance students’ conceptual understanding.

Teachers frequently face barriers such as inadequate training, scarce resources, and insufficient institutional support when attempting to apply PjBL effectively. Problems linked to project-based learning should be taken into consideration and solutions. These include encouraging students to complete their projects, optimizing the teacher’s role as supervisor, schools to finance the students’ projects, selecting projects suitable for the available resources, and facilitating teachers in their daily activities related to Project-based learning ( Cintang et al., 2018). Project-based learning highlights a burden for teachers, but as an effective way to implement a student-centered approach.

The reviewed literature showed that PjBL is mostly applied in physics and mathematics. These results reflect the role of mathematics and physics across STEM disciplines, which emphases on problem-solving and analytical skills ( Wakumire et al., 2022). The reviewed literature aligns with existing literature that recognizes project-based learning is suitable to be applied while teaching and learning subjects requiring critical thinking and practical application ( Abidin et al., 2020). However, the reviewed literature showed that arts, technology, and engineering are explored at a low level. These findings may be due to the insufficiency of laboratory tool kits and curricular structures.

While traditional project-based learning promotes constructivist learning and mastery of disciplinary content, the trending PjBL perspectives should emphasize learning objectives over real-world or community-based problems ( Luque-González et al., 2025). Emerging methodologies, such as socioformative projects and STEAM-based projects, offer more contemporary alternatives ( Tobon & Lozano-Salmorán, 2024). Socioformative projects, for example, focus on action research within communities, fostering interdisciplinary problem-solving and sustainable social development, while STEAM projects integrate creativity and cross-disciplinary collaboration ( Guerra-Macías & Tobón, 2025). The applied methodologies in the reviewed studies of PjBL learning model failed today because it focuses on the “product” or “deliverable,” which can be generated by AI. In line with 21st-century requirements. Effective learning projects should emphasize social-emotional and transferable skills development. We agree with Guerra-Macías and Tobón (2025), who declared that socioformative project-based pedagogical practices are key for academic performance through transversal skills. Thus, employing socioformative pedagogical approaches can contribute to the development of transversal skills and academic performance. Incorporating these approaches highlights potential directions for PjBL to evolve, aligning it with the challenges of the AI era and the development of socially responsible competencies.

In the implementation of the project-based learning (PjBL) model, several structured steps guide the learning process, especially when supported by artificial intelligence (AI). To implement projects, students apply mathematical concepts with the aid of AI tools to get innovative solutions or products. Then, both teachers and students reflect and evaluate their learning experiences and outcomes to reinforce their conceptual understanding ( Sayyid & Rahmatullah, 2025). However, few of the reviewed studies involved automated activities that can be generated by AI. For instance, Santos et al. (2026)’s results showed that GAI with computational and dynamic geometry tools provides patterns of mathematical correctness, justifying classroom-oriented task types in mathematics. The more recent methodologies, like socioformative projects ( Flores & Esteva, 2025) may be effective since they do not focus on the final product, but on ethical interaction with the community, the ethical life project, and face-to-face collaborative work to solve local needs, competencies that AI does not possess.

The reviewed literature highlighted challenges linked to the incorporation of PjBL in education. Lack of support, the teachers’ reluctance, and lack of enough confidence to use PBL, difficulty in time management, and the context where PjBL is implemented, designing reliable assessment tools, played a fundamental role in hindering the effective use of PjBL effectively ( Aldabbus, 2018) indicating an area where more support is needed. It was first suggested that the culture of using PjBL should be spread among schools through workshops, seminars, and training sessions. Second, teachers should be given in-service training on how PjBL is applied. Third, the curriculum should be authentic and purposefully designed to be taught by PjBL. Finally, a special budget for projects should be offered by schools.

The duration of PjBL implementation across the reviewed literature varies considerably depending on the research design and educational context, ranging from short-term instructional units to multi-year longitudinal interventions. Experimental and quasi-experimental studies, such as those by Tika and Agustiana (2021), Twahirwa et al. (2021), and Demir and Önal (2021), generally implemented PjBL over a limited instructional period, typically a few weeks to one academic term. The authors focused primarily on immediate impacts on academic achievement and attitudes. In contrast, the longitudinal study by Han et al. (2016) extended PBL implementation across three consecutive academic years (2008–2010), allowing for sustained exposure and stronger effect sizes in mathematics achievement. Also, Kricsfalusy et al. (2018) examined PjBL within semester-long integration embedded in a programmatic curriculum rather than a short intervention. More recent AI-enhanced PBL studies, such as Ruiz Viruel et al. (2025) and Wan (2025), also appear to follow controlled experimental durations aligned with academic terms. While most studies employed short- to medium-term PjBL implementations. Only a few adopted extended or longitudinal designs, suggesting that sustained, multi-year evaluation of PjBL remains moderately limited in the literature.

## Conclusion

This review underscores the substantial benefits of PjBL in enhancing learning outcomes in line with socioformation. Socioformative pedagogical practices are influenced by socio-emotional skills; the skills needed to be cautiously developed in the 21st-century era. In this regard, enhancing socio-emotional skills is essential for improved student performance. Socioformative projects can extend PjBL beyond classroom achievement by emphasizing community-based problem solving, ethical responsibility, and sustainable development, thereby fostering socially responsive competencies alongside academic learning. Socioformative projects involve flexible proposals centered on critical and socio-critical thinking to contribute to sustainable social development, featuring interdisciplinarity and a high degree of student agency. It is through social learning projects that students communicate, collaborate, and interact with their peers and teachers in a social setting. Such kinds of projects will enhance transferable skills in students, the skills which AI aided projects fail to develop.

Based on the reviewed evidence, this study concludes that project-based learning shows promising potential to support students’ engagement, skill development, and conceptual understanding of STEAM subjects. The findings indicate positive tendencies of universal effects and success. Despite the limited reviewed studies, PjBL appears to be effective if factors such as implementation quality, curriculum alignment, teacher preparedness, and resource availability are observed. However, this review acknowledges persistent challenges that must be addressed to effectively apply PjBL in STEAM education. Given the limited number of studies and the underrepresentation of socioformative applications across STEAM subjects, further rigorous and context-sensitive research is needed to generalize PjBL’s overall effectiveness. In addition, future studies should expand into underexplored subjects for diverse educational settings to build a more comprehensive evidence base.

## Declarations and statements

### Ethics and consent

No ethical approval or consent required.

## Data Availability

No underlying data associated with this article. Figshare: [Project-Based Learning in STEAM subjects for socioformation: a systematic review of contributions, prevalence, and challenges]: 10.6084/m9.figshare.30173854.v4 This project contains the following extended data:
○
Table 1_Review protocol.docx ○
Table 2_Reviewed studies.docx ○ Table 3_Risk of Bias in Systematic Review ○ Table 4_PRISMA 2020 checklist.docx Data are available under the terms of the Creative Commons 1.0 Universal License (CC0 1.0). Figshare: PRISMA checklist for [Project-Based Learning in STEAM subjects for socioformation: a systematic review of contributions, prevalence, and challenges]. 10.6084/m9.figshare.30173854.v4 ( Ukobizaba et al., 2025). Data are available under the terms of the Creative Commons Zero “No rights reserved” data waiver (CC0 1.0 Public domain dedication).
