# Supplementary material for: Project-based learning in STEAM subjects for socioformation: A systematic review of contributions, prevalence, and challenges
Source: F1000Res. 2026 Jun 9;14:1061. Originally published 2025 Oct 10. [Version 3] doi: 10.12688/f1000research.170698.3 (PMC13139846; doi:10.12688/f1000research.170698.3)
Supplement: Peer review history

# Reviewer report: Reviewer response for version 3

**DOI:** 10.5256/f1000research.201824.r492369

**Published:** 2026-06-15

**Authors:** Harlinda Syofyan

**Affiliations:** 1 Universitas Esa Unggul, Jakarta, Indonesia

Okay.

 I have no further comments. Approved.

Are the rationale for, and objectives of, the Systematic Review clearly stated?

Yes

Is the statistical analysis and its interpretation appropriate?

Partly

If this is a Living Systematic Review, is the ‘living’ method appropriate and is the search schedule clearly defined and justified? (‘Living Systematic Review’ or a variation of this term should be included in the title.)

Partly

Are sufficient details of the methods and analysis provided to allow replication by others?

Partly

Are the conclusions drawn adequately supported by the results presented in the review?

Partly

Reviewer Expertise:

Teaching and learning; teaching evaluation; natural science learning;  action reaseach; learning strategy; learning innovation

I confirm that I have read this submission and believe that I have an appropriate level of expertise to confirm that it is of an acceptable scientific standard.

# Reviewer report: Reviewer response for version 2

**DOI:** 10.5256/f1000research.197159.r473585

**Published:** 2026-05-04

**Authors:** Outi Haatainen

**Affiliations:** 1 University of Helsinki, Helsinki, Finland

Dear authors,

 Thank you for the revised version of the manuscript. The study addresses a relevant topic in STEAM education, and the use of a PRISMA-guided systematic review is appropriate for the research questions. I also wish to acknowledge that the revisions made since the first review round, particularly to the methodology section, have improved the overall quality of your manuscript.

 I have some remaining suggestions. Few of the minor and few important for the coherence, and scholarly contribution of the study.

- I recommend mentioning PRISMA already in the abstract to improve methodological clarity for readers from the start.
- Socioformation is a key conceptual foundation of the study, therefore, it should be defined more clearly and explicitly already in the introduction.
- Some claims used to justify the study in the introduction require stronger empirical support. For example, statements regarding the widely recognized impact of PjBL on achievement in mathematics and science, and the limitations of traditional PjBL approaches, should be supported with appropriate citations.
- Related concepts, such as socio-scientific issues (SSI) and other community- or real world problem-oriented frameworks, are widely used in the literature. As these do not appear to be included in the search criteria, it is possible that some relevant studies were not captured. I recommend acknowledging this explicitly as a limitation of the review.
- Figure 2 currently appears to be an incorrect table, as it refers to the number of pathogens rather than the prevalence of PjBL in STEAM subjects. In addition, there is an inconsistency between the results and discussion sections regarding this data (the subjects in which PjBL is most prevalent). Result state sciences versus physics in discussion. I highly recommend rechecking the data, correcting the figure, and ensuring full alignment between results and discussion.

I hope you find these suggestions helpful, and I look forward to seeing the final version.

Are the rationale for, and objectives of, the Systematic Review clearly stated?

Yes

Is the statistical analysis and its interpretation appropriate?

Yes

If this is a Living Systematic Review, is the ‘living’ method appropriate and is the search schedule clearly defined and justified? (‘Living Systematic Review’ or a variation of this term should be included in the title.)

Not applicable

Are sufficient details of the methods and analysis provided to allow replication by others?

Yes

Are the conclusions drawn adequately supported by the results presented in the review?

Partly

Reviewer Expertise:

STEM/STEAM education, project-based learning

I confirm that I have read this submission and believe that I have an appropriate level of expertise to confirm that it is of an acceptable scientific standard, however I have significant reservations, as outlined above.

# Response

**Published:** 2026-05-16

**Authors:** Fidele Ukobizaba

**Affiliations:** University of Rwanda College of Education (URCE), Rwanda

Dear Reviewer,

 Thank you for your input and constructive feedback. The following are responses to the comments provided:

 1) The PRISMA framework was explicitly mentioned in the abstract to provide a clearer methodological orientation from the outset of the study (Kindly see the abstract section).

 2) Socioformation, which serves as a key conceptual foundation of the research, was more clearly and explicitly explained in the introduction section (Kindly see the introduction section).

 3) Several claims in the introduction were strengthened through the inclusion of appropriate supporting citations to improve academic rigor (Kindly see the introduction section).

 4) The socio-scientific issues (SSI) and other community- or real-world problem-oriented frameworks were mentioned as a limitation (Kindly see the conclusion section).

 5) Figure 2 was provided during the submission as a pie chart illustrating the prevalence of Project-Based Learning (PjBL) across STEAM subjects. Finally, the discussion and interpretation of the results, particularly those related to prevalence, were refined and expanded to improve clarity, coherence, and depth of analysis (Kindly see the discussion).

 Sincerely,

# Reviewer report: Reviewer response for version 2

**DOI:** 10.5256/f1000research.197159.r471983

**Published:** 2026-04-08

**Authors:** Harlinda Syofyan

**Affiliations:** 1 Universitas Esa Unggul, Jakarta, Indonesia

The revised manuscript shows clear and meaningful improvement. The authors have adequately addressed the previous comments, particularly in strengthening the methodology, clarifying the data analysis, and improving the consistency of the results and discussion.

 Overall, the revisions have enhanced the clarity, rigor, and coherence of the study. The manuscript is now considered acceptable for Indexing.

Are the rationale for, and objectives of, the Systematic Review clearly stated?

Yes

Is the statistical analysis and its interpretation appropriate?

Partly

If this is a Living Systematic Review, is the ‘living’ method appropriate and is the search schedule clearly defined and justified? (‘Living Systematic Review’ or a variation of this term should be included in the title.)

Partly

Are sufficient details of the methods and analysis provided to allow replication by others?

Partly

Are the conclusions drawn adequately supported by the results presented in the review?

Partly

Reviewer Expertise:

Teaching and learning; teaching evaluation; natural science learning;  action reaseach; learning strategy; learning innovation

I confirm that I have read this submission and believe that I have an appropriate level of expertise to confirm that it is of an acceptable scientific standard.

# Reviewer report: Reviewer response for version 1

**DOI:** 10.5256/f1000research.188189.r446299

**Published:** 2026-01-27

**Authors:** Sergio Tobón

**Affiliations:** 1 Centro Universitario CIFE, Cuernavaca, Mexico; 2 Universidad Aoma de Chihuahua, Chihuahua, Chihuahua, Mexico

"Project-based learning in mathematics and science: a review of contributions, prevalence, and challenges"

**Originality and Relevance:** The article addresses a relevant topic, but its approach is traditional and lacks originality in its current state. By relying exclusively on constructivism and classic PBL, it overlooks emerging pedagogical trends necessary to face the era of Artificial Intelligence (AI), such as socioformation.

**Language:** The English text contains grammatical errors and typos (e.g., "pedagocy", "studeis") that require professional editing.

**Suggestions for Improvement:**

**1. Title:** Update the title to reflect a critical perspective regarding current challenges and specify that this is a systematic review.

**2. Abstract:** Restructure the abstract to strictly follow the IMRaD format and address the gap regarding AI and sustainable social development.

**Introduction:**

**3. Moving Beyond Constructivism:** The text grounds PBL exclusively in constructivist theories where students "construct knowledge." It fails to consider other, more current and pertinent approaches. I suggest considering new theories or pedagogical models such as socioformation. Constructivism is insufficient in the AI era, as algorithms can "construct" and organize information faster than humans.

**4. Other More Current and Relevant Project Methodologies:** Furthermore, you must analyze the shortcomings of traditional project-based learning methodology, which tends to focus more on learning objectives. Consider other methodologies such as socioformative projects and STEAM. Socioformative projects, for example, focus on action research within a territory and sustainable social development through an interdisciplinary approach. This is more relevant for facing the challenges of artificial intelligence. Traditional PBL in science tends to be disciplinary, focused more on content and learning processes than on real community problems.

**5. Research Gap or Problem Statement:** Expand the problem statement to analyze potential issues, failures, or drawbacks of project-based learning, and consider recent alternative methodologies that are more pertinent to the AI era.

**6. Research Questions:** Refine the research questions to better address the knowledge gap. Consider integrating the suggestions provided to broaden the scope of analysis.

**7. Methodology:** Expand the sample size and search criteria. Analyzing only 20 studies is very limited for a systematic review, especially considering the inclusion of sciences. **Suggestion:** Broaden the search by including keywords such as "Socioformative projects," "formative projects," "STEAM education," "AI in PBL," "Sustainability competencies," "socioformation," and "sustainable social development."

**8. Results:** It is necessary to expand the analysis and focus on a deep critical review. I suggest not limiting the results to simply listing which subjects use PBL.

- Evaluate whether the PBL methodology employed focuses on achieving learning processes (constructivist approach) or truly addresses environmental or territorial issues (socioformation).
- Analyze whether the methodologies treat problems as mere excuses to learn (constructivism) or view the problems as a key purpose of the training itself (socioformation).
- Analyze the extent to which the project-based learning focuses on academic performance versus a commitment to transforming environments (socioformative pedagogical model).
- Review the extent to which the use of PBL generates activities and products that are automatable in the AI era, and the extent to which non-automatizable processes are addressed, such as an emphasis on critical and socio-critical thinking, ethics, and awareness of sustainable social development.

**Discussion:**

**9. Critical Analysis of PBL Shortcomings Regarding Artificial Intelligence:** The article omits AI. It must discuss how traditional PBL fails today because it focuses on the "product" or "deliverable," which can be generated by AI. Consider proposing more recent methodologies like socioformative projects, where the focus is not on the final product, but on ethical interaction with the community, the ethical life project, and face-to-face collaborative work to solve local needs—competencies that AI does not possess.

**10. Contrast of Pedagogical Models:** Replace the validation of constructivist "learning by doing" with research and problem-solving within a territory or context from the perspective of socioformation. Explain that while constructivism seeks individual learning, socioformation seeks service and environmental improvement, shielding the educational process against uncritical automation. Consider presenting more flexible proposals centered on critical and socio-critical thinking to contribute to sustainable social development, featuring interdisciplinarity and a high degree of student agency.

Are the rationale for, and objectives of, the Systematic Review clearly stated?

Partly

Is the statistical analysis and its interpretation appropriate?

Not applicable

If this is a Living Systematic Review, is the ‘living’ method appropriate and is the search schedule clearly defined and justified? (‘Living Systematic Review’ or a variation of this term should be included in the title.)

No

Are sufficient details of the methods and analysis provided to allow replication by others?

Partly

Are the conclusions drawn adequately supported by the results presented in the review?

Yes

Reviewer Expertise:

My areas of expertise are project-based work in education and the study of educational and pedagogical models

I confirm that I have read this submission and believe that I have an appropriate level of expertise to confirm that it is of an acceptable scientific standard, however I have significant reservations, as outlined above.

## References

[1] : Socioformative pedagogical practices and academic performance in students: Mediation of socioemotional skills. *Heliyon* .2024;10(15) : 10.1016/j.heliyon.2024.e34898 10.1016/j.heliyon.2024.e34898 PMC1133628639170493

[2] : Development of transversal skills in higher education programs in conjunction with online learning: relationship between learning strategies, project-based pedagogical practices, e-learning platforms, and academic performance. *Heliyon* .2025;11(2) : 10.1016/j.heliyon.2024.e41099 10.1016/j.heliyon.2024.e41099 PMC1178665039897793

# Response

**Published:** 2026-02-25

**Authors:** Fidele Ukobizaba

**Affiliations:** University of Rwanda College of Education (URCE), Rwanda

We sincerely thank the reviewer for the constructive and thought-provoking comments. Each suggestion has been carefully considered, and the manuscript has been substantially revised as detailed below.

 Regarding “originality and relevance”, the reviewer noted that the manuscript relied excessively on constructivism and traditional PBL, overlooking emerging pedagogical trends such as socioformation in the AI era. In response, we expanded the theoretical framework to integrate socioformation alongside constructivism. The Introduction (pp. 2–4) now explains the limitations of a purely constructivist perspective and introduces socioformation as a model oriented toward ethical life projects, territorial problem-solving, and sustainable social development. Additionally, references to Artificial Intelligence (AI) and its educational implications were incorporated throughout the manuscript, including the Results section under “Enhancing students’ skills for socioformation” (p. 9).

 Concerning “language quality,” the entire manuscript was thoroughly revised to correct grammatical errors and typographical mistakes (e.g., “pedagocy,” “studeis”). These corrections were implemented consistently across all sections.

 With respect to the “title:, it was revised to better reflect the critical scope and systematic nature of the study. The updated title " *Project-based learning in STEAM subjects for socioformation: a systematic review of contributions, prevalence, and challenges" *explicitly emphasizes both socioformation and the systematic review design.

 The “abstract” was restructured strictly according to the IMRaD format. The revised version clearly presents Background, Methods, Results, and Conclusions, and explicitly addresses the research gap concerning AI and sustainable social development (see Abstract section).

 In response to the recommendation to move beyond constructivism, the “Introduction” (pp. 2–5) now contrasts constructivist learning theory with socioformation. The fifth paragraph (p. 5) explicitly discusses why traditional PBL models centered on content acquisition are insufficient in the AI era.

 The reviewer’s suggestion to analyze other relevant project methodologies was addressed by examining socioformative projects and interdisciplinary STEAM approaches in the Introduction (p. 5). The shortcomings of traditional PjBL, particularly its disciplinary focus and emphasis on deliverables, are now critically analyzed.

 The “research gap and problem statement” was expanded (p. 5) to highlight limitations of traditional PBL and justify the inclusion of socioformative methodologies. Consequently, the research questions were refined (p. 4) to reflect this broader and more critical analytical framework.

 Regarding the “methodology”, the sample size was expanded from 20 to 32 studies through a deeper search strategy using broader keyword combinations related to socioformation, sustainability, and AI (see Methodology section).

 The “Results section” was substantially deepened. Rather than merely listing subject areas, it now critically evaluates whether reviewed studies emphasize constructivist learning processes or socioformative territorial transformation. See “Enhancing students’ engagement for socioformation” (p. 8), “Enhancing students’ creativity for socioformation” (p. 9), and “Academic performance for environment transformation” (p. 10). The analysis also distinguishes between automatable outputs and non-automatizable competencies such as ethical reasoning and socio-critical thinking.

 In the “Discussion” (pp. 15–17), a critical analysis of traditional PBL in the AI era was added (seventh to ninth paragraphs, pp. 16–17). The manuscript now explains how product-oriented PBL may be vulnerable to AI automation, whereas socioformative pedagogies emphasize ethical community engagement and face-to-face collaborative problem-solving. Furthermore, a clear contrast between constructivist and socioformative pedagogical models was incorporated (seventh paragraph, pp. 15–16), highlighting the shift from individual knowledge construction to service-oriented, interdisciplinary, and sustainability-focused education.

# Reviewer report: Reviewer response for version 1

**DOI:** 10.5256/f1000research.188189.r423370

**Published:** 2025-10-27

**Authors:** Harlinda Syofyan

**Affiliations:** 1 Universitas Esa Unggul, Jakarta, Indonesia

**1. Methodology**

1. Search criteria are less specific: Search keywords are too generic. Add more systematic keyword combinations using Boolean operators (AND, OR, NOT).
2. Publication time span is too wide (2000-2023): Consider focusing on the last 10 years to get findings that are more relevant to the current educational context.
3. Risk of bias assessment is mentioned but the results are not explained in detail. Add a more comprehensive table or explanation.

**2. Data Analysis**

1. Only 20 articles out of 202 were used - this ratio is very small (9.9%). Need a more detailed explanation of the exclusion criteria.
2. No quantitative meta-analysis: If possible, add effect size or aggregate statistics from the studies reviewed.
3. The categorization of the study (3 for engagement, 8 for skills, 9 for performance) overlaps and needs to be clarified.

**3. Results and Discussion**

1. The prevalence data (Figure 2) does not have an explanation of how the percentages are calculated. Was it from the 20 studies reviewed or other sources?
2. Contradictions are not discussed in depth: Holmes & Hwang's (2016) studies that found no significant differences need further analysis – what distinguishes their context?
3. Overgeneralization: Conclusions are too general when only based on 20 studies.

**4. Technical Weaknesses**

1. Inkonsistensi citation style: some use "eg." some use  "e.g."
2. Typo: "BjBL was fought" should "thought" (page 7)
3. Figure 1 (PRISMA diagram) - The numbers are inconsistent - need to recheck the exclusion flow
4. Tidak ada informasi About Inter-rater Reliability in the Article Selection Process

**5. Contextual Gaps**

1. The geographical context is unclear: From which countries are these studies from? Can the findings be generalized to the Rwanda/Africa context?
2. Education level is not consistently specified (elementary, junior high, high school mixed)
3. The duration of PjBL implementation in the reviewed studies was not discussed

Are the rationale for, and objectives of, the Systematic Review clearly stated?

Yes

Is the statistical analysis and its interpretation appropriate?

Partly

If this is a Living Systematic Review, is the ‘living’ method appropriate and is the search schedule clearly defined and justified? (‘Living Systematic Review’ or a variation of this term should be included in the title.)

Partly

Are sufficient details of the methods and analysis provided to allow replication by others?

Partly

Are the conclusions drawn adequately supported by the results presented in the review?

Partly

Reviewer Expertise:

Teaching and learning; teaching evaluation; natural science learning;  action reaseach; learning strategy; learning innovation

I confirm that I have read this submission and believe that I have an appropriate level of expertise to confirm that it is of an acceptable scientific standard, however I have significant reservations, as outlined above.

# Response

**Published:** 2026-02-25

**Authors:** Fidele Ukobizaba

**Affiliations:** University of Rwanda College of Education (URCE), Rwanda

**1. Methodology**

 Regarding the search criteria, the initial keywords were considered too generic. In response, the search strategy was refined by incorporating more systematic keyword combinations using Boolean operators (AND, OR, NOT). This ensured greater precision and reproducibility in the identification of relevant studies (see Methodology section, first paragraph, p. 4).

 Concerning the wide publication time span (2000–2023), the review period was narrowed to 2015–2026 to better reflect contemporary educational contexts and recent developments in STEAM education. This adjustment strengthens the relevance of the findings (see

 Methodology section, second paragraph, p. 5).

 The comment on insufficient explanation of risk of bias was addressed by applying the ROBIS protocol. A clearer explanation of the assessment process and its outcomes was added, indicating an overall low risk of bias (see Methodology section, pp. 5–6).

**2. Data Analysis**

 The concern about the small proportion of included studies (20 out of 202) was addressed by conducting a deeper search with expanded keywords and Boolean combinations. This increased the final sample to 32 studies, and exclusion criteria were clarified (see Table 2 in the Figshare repository).

 A quantitative meta-analysis was not added due to significant heterogeneity across study designs, contexts, and reported outcomes, which made effect size aggregation methodologically inappropriate.

 To resolve overlap in study categorization (engagement, skills, performance), clearer definitions and classification criteria were provided to avoid redundancy (see Methodology section, pp. 5–6).

**3. Results and Discussion**

 The calculation of prevalence percentages in Figures 2–4 was clarified. Percentages were recalculated based on the updated sample of 32 reviewed studies and explicitly explained in the text.

 Regarding the alleged contradiction in Holmes and Hwang (2016), a re-examination confirmed no inconsistency with the overall findings; therefore, no additional contradiction analysis was included.

 To address overgeneralization, the conclusion was revised to align strictly with evidence derived from the 32 reviewed studies (see Conclusion, p. 17).

**4. Technical Weaknesses**

 Citation inconsistencies were corrected by standardizing the use of “e.g.” throughout the manuscript. Typographical errors, including “BjBL was fought,” were corrected. The PRISMA diagram was revised to ensure numerical consistency in the inclusion–exclusion flow (see Figure 1). Additionally, information on inter-rater reliability (IRR) in the article selection process was added (see Methodology section, p. 5).

**5. Contextual Gaps**

 The geographical distribution of studies was analyzed and explicitly reported and analyzed. (see Results, pp. 12–13). Education levels (elementary, junior, senior high) were systematically identified and reported (see Results, p. 12). Finally, the duration of PjBL implementation across studies was examined and discussed to enhance contextual depth (see Discussion, pp. 16–17).
